# Temporal Changes in the Skin Microbiome of Epidermolysis Bullosa Patients following the Application of Wound Dressings

**DOI:** 10.3390/jcm12206435

**Published:** 2023-10-10

**Authors:** Amir Horev, Michael Brandwein, Avraham Vaknin, Yair Motro, Jacob Moran-Gilad

**Affiliations:** 1 Pediatric Dermatology Service, Soroka University Medical Center, Yitzhak Rager Ave., P.O. Box 151, Beer Sheva 8410101, Israel; 2 Faculty of Health Sciences, Ben-Gurion University of the Negev, Beer Sheva 8410101, Israel; michael.brandwein@mail.huji.ac.il (M.B.); motroy@post.bgu.ac.il (Y.M.); giladko@post.bgu.ac.il (J.M.-G.); 3 Department of Pediatrics, Soroka University Medical Center, Beer Sheva 8410101, Israel; avrahamva@clalit.org.il

**Keywords:** Epidermolysis bullosa, skin microbiome, children, 16S *rRNA* amplicon sequencing

## Abstract

Objective: Epidermolysis bullosa (EB) is a group of rare hereditary skin disorders characterized by the formation of painful blisters, erosions, and ulcers. In addition, the wounds can easily become infected with different pathogens. Therefore, the dynamics in the microbial populations across the various stages of EB can shed light on pathophysiology, the effect of treatment, and the factors involved in its recovery, but they are understudied. We thus sought to characterize the skin microbiome among patients with EB over time. Methods: A prospective study conducted in the pediatric dermatology clinic at Soroka Medical Center, Beer-Sheva, Israel. Children (0–18) with simplex and recessive dystrophic EB were sampled at two different time points: before a therapeutic regimen and 90 days (±14 days) later. Samples were obtained from lesional skin (wound), healthy, non-lesional skin, and seborrheic skin (forehead). Samples were subject to 16S *rRNA* amplicon sequencing. Analyses performed included comparisons of relative abundance at the phyla and genera taxonomic levels, alpha and beta diversity comparisons, and differential abundance. Results: 32 children with EB were enrolled, for whom 192 skin microbiome samples were obtained. Lesional skin samples harbored significantly less *Bacteroidota* and *Fusobacteriota* before the initiation of treatment. Following topical dressing, we observed more *Firmicutes* and less *Proteobacteria* in lesional skin samples than healthy and seborrheic skin samples. In addition, *Staphylococcus* was significantly more abundant in lesional samples than in non-lesional and seborrheic samples following treatment. Conclusions: Our study recaptured the reduced bacterial diversity and increased staphylococcal carriage in EB patients, showing a potential effect of topical dressing either directly on the wound microbiome or indirectly through the contribution towards skin healing. The detection of *Firmicutes* in general, and *S. aureus* specifically, commensurate with the application of a wound dressing may warrant the use of additional treatment methods to facilitate wound healing. Future studies in these patients should prospectively correlate the temporal changes in the microbiome associated with various treatment modalities in order to optimize the care of EB patients.

## 1. Introduction

Epidermolysis bullosa (EB) is a heterogeneous group of rare, genetic skin disorders caused by gene mutations that encode the proteins that are part of the hemidesmosomes and focal adhesion complex [1]. There are four major EB types: simplex, junctional, dystrophic, and Kindler. EB is characterized by the weakening of the skin and mucous tissues, leading to painful erosions and blisters of the skin and mucous membranes following minor friction or trauma. The wounds can easily become infected with pathogens such as *Staphylococcus* spp. Furthermore, the frequent use of antibiotics in these patients, sometimes in combination, and for longer duration, may select for antibiotic-resistant strains [2]. Moreover, the incidence of resistant skin bacteria, such as methicillin-resistant *S. aureus* (MRSA) is rising globally and their role in skin infections is becoming more prominent, thus impacting the management of skin disorders. Chronic erosions and ulcers such as those seen in EB may be colonized by commensal microorganisms, contributing to worsening inflammatory conditions and decreased wound healing [3].

Studies using 16S *rRNA* amplicon sequencing have consistently found that skin bacteria mainly belong to four major phyla: *Actinobacteria*, *Firmicutes*, *Bacteroidetes*, and *Proteobacteria*. The relative abundance of different taxa varies among different skin sites and between healthy or diseased skin [4,5]. Dramatic temporal changes in the human skin microbiome in the absence of severe external perturbances are rare [3]. A comprehensive characterization of the dynamics in the microbial populations across the various stages of the inflammatory process in EB can contribute to understanding the disease’s pathophysiology, the treatment’s effect, and the factors involved in its recovery.

Despite the growing body of literature on the skin microbiota in health and disease, there is a paucity of data concerning the microbiome features of EB. Therefore, in an effort to shed light and characterize the skin microbiome in EB and understand its role, we studied the skin microbiome of individuals with EB over time.

## 2. Patients and Methods

### 2.1. Study Design

A prospective study was conducted to characterize the skin microbiome of pediatric EB patients. The study was approved by the Ethics Review Committee of the Soroka University Medical Center (SUMC), Beer-Sheva Israel (protocol #0391-15-SOR). This study was conducted in accordance with the Declaration of Helsinki and all appropriate amendments. Written informed consent was obtained from one or both parents of each patient before entering the study. Patients were sampled at two separate time points: before beginning the therapeutic regimen and again 90 days (±14 days) later.

### 2.2. Study Population

Patients were enrolled in the pediatric dermatology clinic at SUMC between January and November 2021. Children (≤18 years) diagnosed with localized and generalized simplex or recessive dystrophic EB were included in the study. Exclusion criteria included pregnancy and the use of systemic or topical antibiotics in the month preceding study enrollment. Our study attempted to enroll all EB patients followed in this clinic.

### 2.3. Sample Collection

Study enrollees were instructed to avoid bathing and emollient treatment in the 12 h preceding sampling. A complete medical history and skin examination was carried out upon enrollment by a physician. Additional essential data collected included date of birth, sex, ethnicity, diagnosis date, family history, previous hospitalizations, history of other illnesses, and previous treatments. Samples were obtained from lesional skin, healthy skin, and seborrheic skin (forehead). Lesional samples were taken primarily from the limb and healthy skin samples were taken preferably from contralateral unaffected skin. When the contralateral skin was lesional, the sample was taken from an unaffected area of skin at least 10 cm away from the first sample. A sterile rayon-tipped swab (Copan, Brescia, Italy) soaked in sterile 0.15 M NaCl with 0.1% Tween 20 (JT Baker, Phillipsburg, NJ, USA) was used to sample skin and a 2 cm × 2 cm area of skin was sampled. Samples were stored at −80 °C until processing. Sample collection was repeated 90 days (±14 days) later from the same locations. Patients were instructed to use non-adhesive dressings such as Vaseline^®^ petrolatum and PolyMem^®^ foam dressing on the lesional skin sampled and were instructed to replace the dressing every 48 h until the area healed.

### 2.4. DNA Extraction and 16S rRNA Gene Amplicon Sequencing

Genomic DNA was extracted from swabs using an LEV blood kit (Promega, Madison, WI, USA) implemented on a Maxwell 16 instrument, following the manufacturer’s protocol with several modifications. Modifications included a lysozyme incubation (10 ng/µL lysozyme; Thermo Fisher Scientific, Waltham, MA, USA) for 30 min at 37 °C, followed by bead-beating (40 s at 6 m/s) using a FastPrep-24 System (MP Biomedicals, Santa Ana, CA, USA). Homogenized samples were transferred to the Maxwell cartridges for the final purification of DNA. Genomic DNA was prepared for sequencing using a two-stage amplicon sequencing workflow, as described previously [6]. Initially, genomic DNA was PCR amplified using primers targeting the V4 region of microbial 16S ribosomal RNA (rRNA) genes. The primers, 515F modified and 806R modified [7], contain 5′ linker sequences compatible with Access Array primers for Illumina sequencers (Fluidigm, South San Francisco, CA, USA). PCRs were performed in a total volume of 10 microliters using MyTaq™ HS 2X Mix (Bioline, London, UK), primers at 500 nM concentration, and approximately 1000 copies per reaction of a synthetic double-stranded DNA template as described previously [8,9]. Thermocycling conditions were as follows: 95 °C for 5′ (initial denaturation), followed by 28 cycles of 95 °C for 30 s, 55° for 45 s, and 72 °C for 30 s. Each reaction’s microliter of PCR product was transferred to the second-stage PCR reaction. Each reaction was conducted in a final volume of 10 microliters using MyTaq HS 2X mix, and each well contained a unique primer pair of Access Array primers containing Illumina sequencing adapters, single index sample-specific barcode, and linker sequences. Thermocycling conditions were as follows: 95 °C for 5′ (initial denaturation), followed by 8 cycles of 95 °C for 30 s, 60° for 30 s, and 72 °C for 30 s. Libraries were pooled and purified using 0.6× concentration of AMPure XP beads (Beckman Coulter, Brea, CA, USA) to remove short fragments below 300 bp. Pooled libraries were loaded onto a MiniSeq sequencer (Illumina, San Diego, CA, USA) with 15% phiX spike-in and paired-end 2 × 153 base sequencing reads. Positive and negative run controls were included.

The synthetic double-stranded DNA spike was synthesized as a gBLOCK by Integrated DNA Technologies (IDT; Coralville, IA, USA). The basis of the design was a 999 bp region of the 16S rRNA gene of *Rhodanobacter denitrificans* strain 2APBS1^T^ (NC_020541; e.g., Kostka et al., 2012 [10]). The V4 variable region sequence was replaced by portions of a eukaryotic messenger RNA sequence (*Strongylocentrotus intermedius* glyceraldehyde-3-phosphate dehydrogenase mRNA; KC775387). Primer sites were preserved, and the synthetic DNA’s overall length (in bp) did not differ from the equivalent fragment of *R. denitrificans*. PCR amplicons generated from this synthetic DNA do not differ in size from bacterial amplicons and can only be identified and removed through post-sequencing bioinformatics analysis.

### 2.5. Data Processing and Analysis

Samples underwent quality control (QC), trimming, merging, and removal of the synthetic DNA template using FastQC [11], Trimmomatic [12], bbmerge, and bbmap (BBTools v37.28) [13], respectively. Forward and reverse reads (i.e., read pairs) were trimmed to remove adapters and low-quality read ends and merged. The sample library size varied from 1 to 88,643 reads, with a mean and median sample library size of 15,871 and 10,321, respectively. Following QC and filtering, the sequenced amplicon reads were imported into the QIIME2 package (v2021.8) and analyzed with the DADA2 pipeline. After denoising, dereplication, and chimera filtering, the resulting amplicon sequence variants (ASVs) counts per sample in the feature table varied from 1 to 37,513, with a mean and median of 9074 and 6709, respectively. Finally, ASV counts were rarefied to 1400 per sample, and the sequences from the remaining 166 rarefied samples were subsequently assigned to taxonomic groups with the SILVA *rRNA* database (SILVA release 132, 99%, with the QIIME2 classifier trained on the 515F/806R V4 region of 16S) [14,15] and used for downstream analyses.

Analyses performed included comparisons of relative abundance at the phyla and genera taxonomic levels, comparisons of alpha and beta diversity, and differential abundance using linear discriminant analysis (LDA) effect size (LefSe) analysis [16]. LefSe results were considered significant if the LDA score was greater than three. The Shannon diversity and Faith’s phylogenetic diversity (PD) indices were used for alpha diversity. The Bray-Curtis and Unweighted Unifrac dissimilarity metrics were used for beta diversity between populations. For alpha diversity, statistical comparisons between two independent groups used the Wilcoxon test, while comparisons between more than two independent groups used the Kruskal-Wallis (KW) test. For beta diversity, statistical analyses were implemented using the PERMANOVA test (with 999 permutations) for overall group comparisons and the post hoc Dunn test for pairwise comparisons. Regarding statistical power, we estimated that for comparison of two sample groups (lesional vs. non-lesional skin) with a sample size of 50 samples in each group, the power for identifying a difference of mean abundance of 50% will be >0.9. All *p*-values were adjusted using the Benjamini-Hochberg (FDR) method. Analyses were conducted and plots were generated using the R package microeco (v0.11.0) [17].

## 3. Results

### 3.1. General Description of the Cohort

We enrolled 32 children with EB, including 20 (62.5%) with localized simplex, eight (25%) with generalized simplex, and four (12.5%) with recessive dystrophic EB. The patients’ ages ranged from 1 month to 16 years old (average of 4.7 years old); 90.6% of patients were Bedouin and 68.7% were male. All dystrophic EB patients had been diagnosed genetically, and the simplex EB patients were diagnosed by clinical or genetic evaluation. The study cohort’s demographic, clinical, and genetic features are presented in Table 1. A total of 192 skin microbiome samples were collected, of which 166 were successfully sequenced, passed QC, and underwent analyses. No patient was under topical or systemic corticosteroid treatment at the time of inclusion.

### 3.2. Taxonomic Abundance

**Figure 1:**
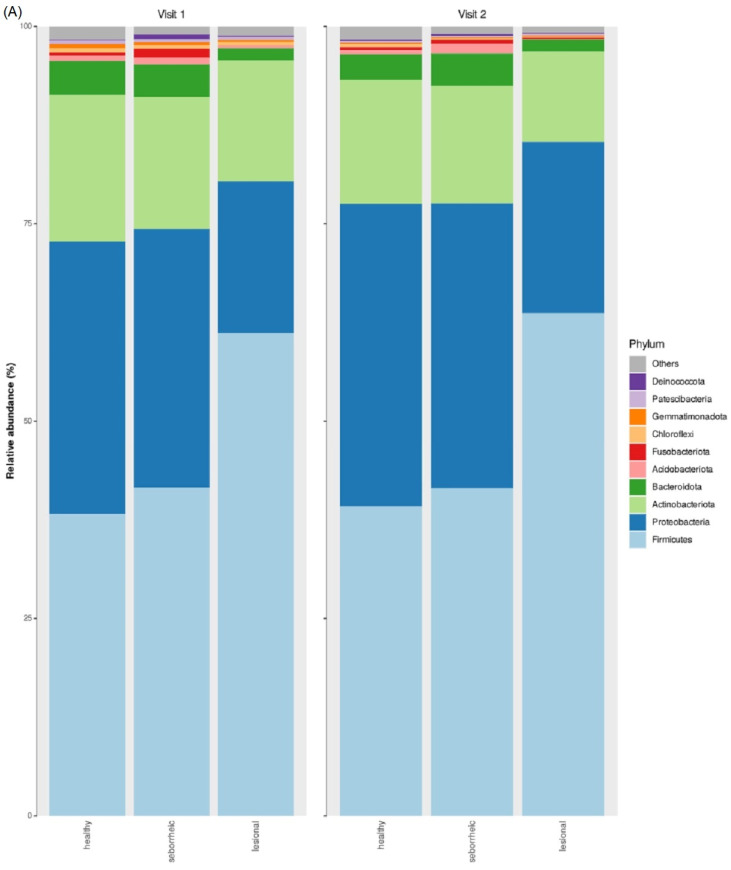

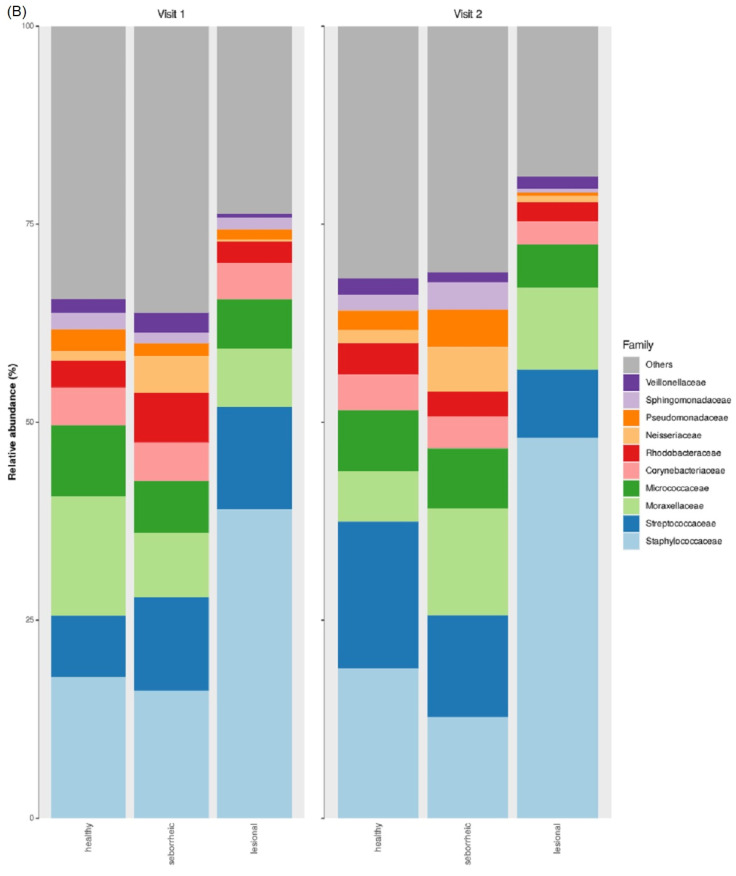

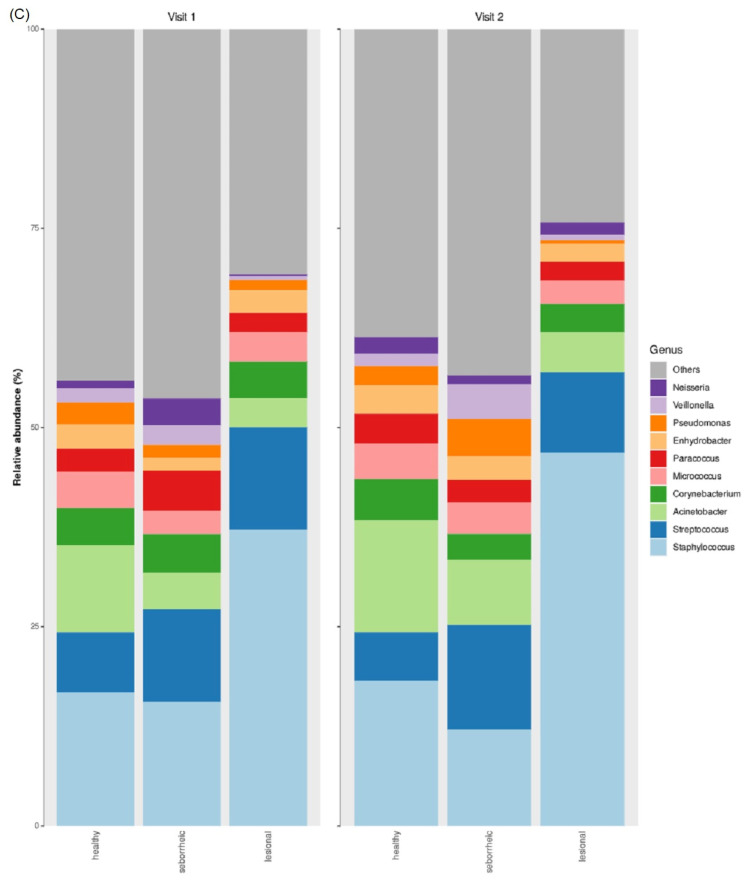

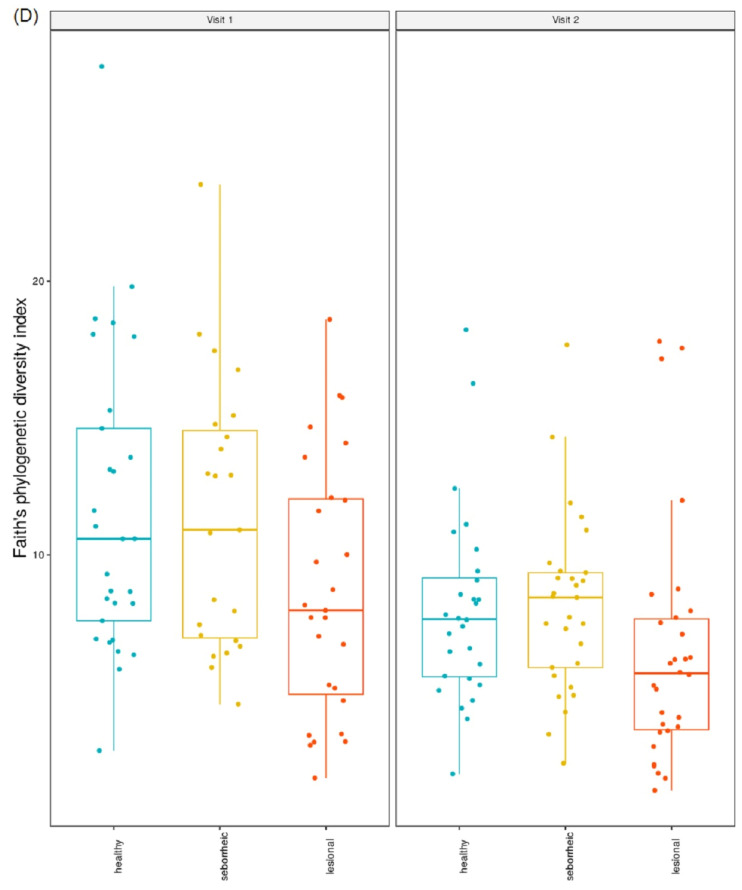

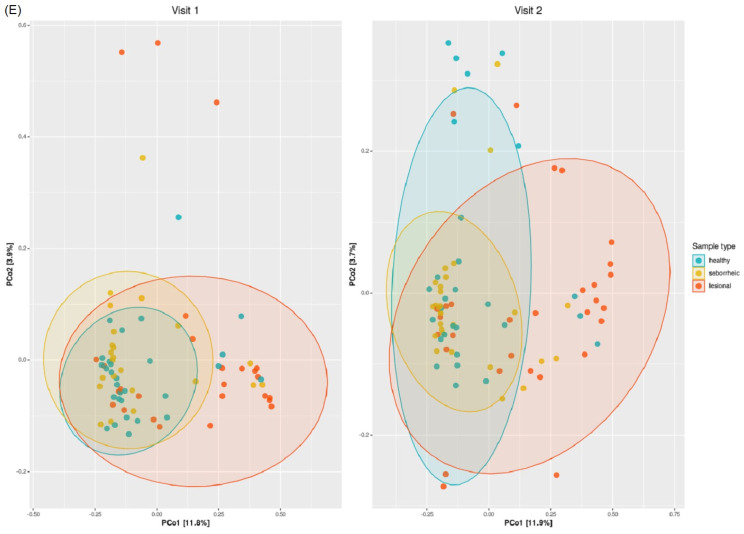

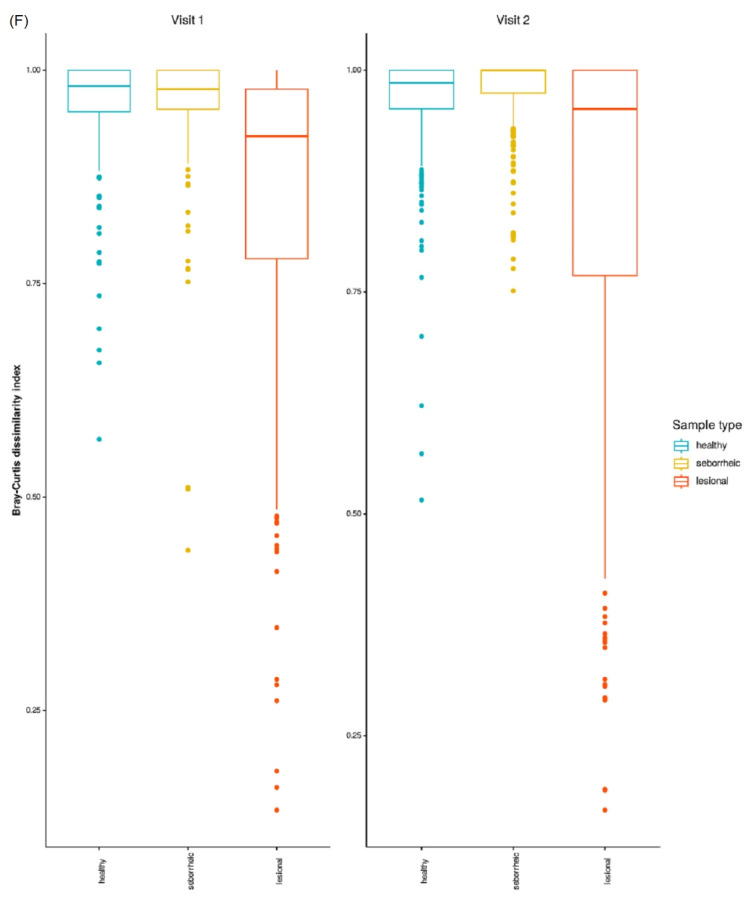
Temporal Dynamics of the Cutaneous EB Microbiome: Mean relative abundances for the top 10 most abundant taxa of healthy (*n* = 29/28), seborrheic (*n* = 23/29), and lesional (*n* = 27/30) sample types at visit 1 and visit 2. (**A**) Phylum taxonomic level; (**B**) Family taxonomic level; (**C**) Genus taxonomic level. Alpha diversity analysis (Faith’s phylogenetic diversity index) for healthy, seborrheic, and lesional samples at visit 1 and visit 2 (**D**). Statistically significant difference was only observed in visit 2 between seborrheic and lesional samples (KW and post hoc Dunn’s test, FDR *p*-value < 0.03). Beta diversity analysis (Bray-Curtis dissimilarity index) for healthy, seborrheic, and lesional samples at visit 1 and visit 2. Principal coordinates analysis (PCoA) plot of sample types at visit 1 and visit 2, with confidence ellipses (**E**). PERMANOVA (999 permutations) between sample types at visit 1 and visit 2 showed statistically significant (FDR *p*-value < 0.05) differences between lesional and healthy samples, and lesional and seborrheic samples at both visits (**F**).

In both visits (i.e., visit 1 and 2), phyla dominant in all sample sites (i.e., healthy, seborrheic, and lesional) included Firmicutes, Proteobacteria, Actinobacteriota, and Bacteroidota (Figure 1A and Figure S1). [4] In both visits, Bacteroidota and Fusobacteriota were significantly less abundant in lesional samples (means 1.5% and 0.04%, respectively) when compared to healthy and seborrheic samples (means 3.7%/4.1% and 0.45%/1.2%, respectively; KW FDR *p*-value < 0.05). In visit 2, Firmicutes were significantly more abundant in lesional samples (mean 62.5%) when compared to healthy and seborrheic samples (means 38.7% and 41.5%, respectively; FDR *p*-value < 0.01), while Proteobacteria were significantly less abundant in lesional samples (mean 20.5%) when compared to healthy and seborrheic samples (means 36.4%/34.6%, respectively; FDR *p*-value < 0.05). These significant differences were also observed in the family (Figure 1B and Figure S2) and genus taxonomic levels (Figure 1C and Figure S3). They included *Neisseriaceae*, *Leptotrichiaceae*, *Staphylococcus*, *Actinomyces*, and *Veillonella* (KW, FDR *p*-value < 0.05).

### 3.3. Alpha Diversity

No significant differences were observed between sample sites (i.e., lesional, healthy, and seborrheic) when compared using the Shannon Diversity index. However, significant differences were observed (Figure 1D) between lesional (mean = 7.5) and both healthy and seborrheic samples (mean = 9.8 and 9.6, respectively) in the second visit, using Faith’s phylogenetic diversity index (KW; FDR *p*-value < 0.03). This indicates that all sample types were rich in bacteria, though lesional skin samples were less phylogenetically diverse.

### 3.4. Beta Diversity

Significant differences were observed (PERMANOVA, 999 permutations, FDR *p*-value < 0.003) between lesional skin samples and both healthy and seborrheic samples in both visits, using the Bray-Curtis dissimilarity measure (Figure 1E). A greater dissimilarity was observed between healthy and seborrheic samples (with mean values at visit 1/visit 2: 0.9655/0.9611 and 0.9658/0.9798, respectively) than with lesional samples (with mean values at visit 1/visit 2: 0.8530/0.8523) (Figure 1F).

### 3.5. Differences in Microbiome Taxonomic Composition Per Sample Type

**Figure 2:**
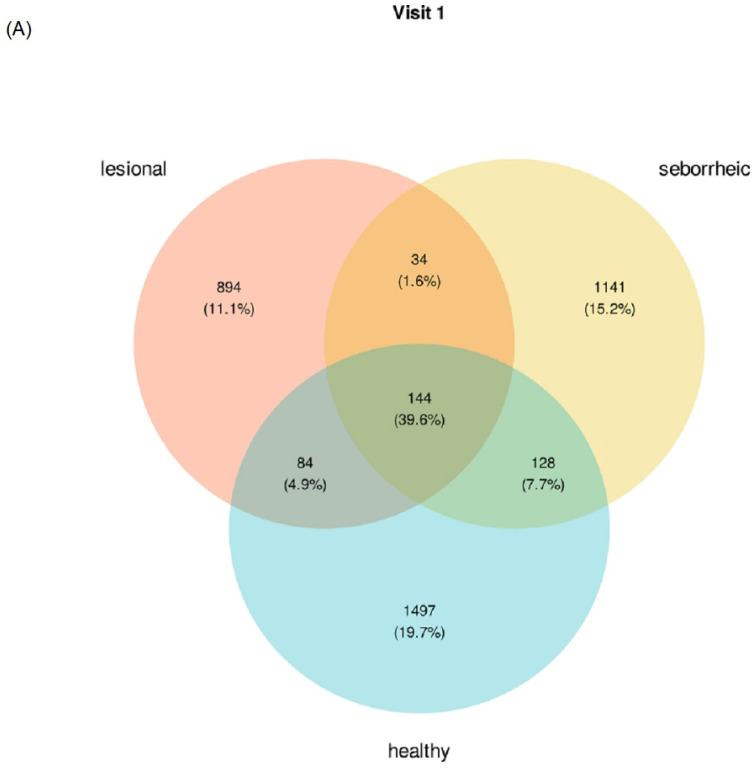

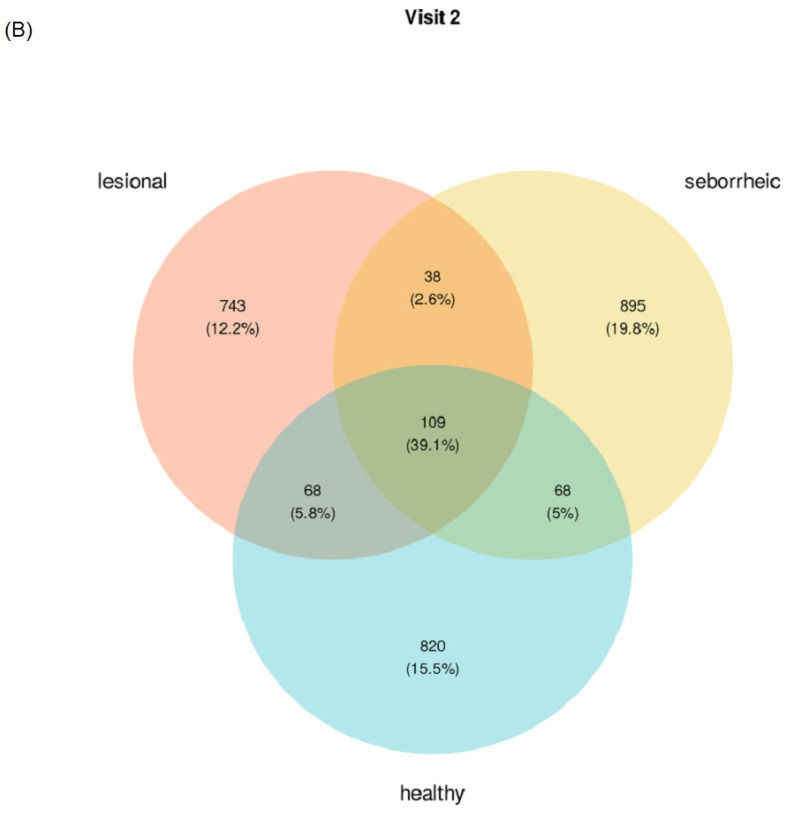

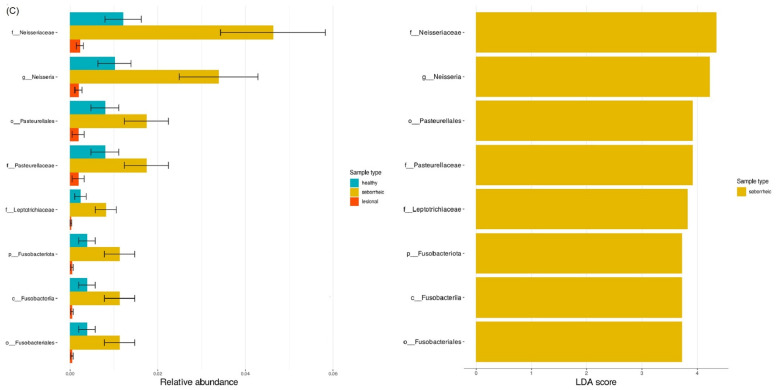
Shared and Unique Microbiome of Different Sample Types: Venn diagram representation of unique and shared amplicon sequence variants (ASVs) between healthy, seborrheic, and lesional sample types at (**A**) visit 1 and (**B**) visit 2. Linear Discriminant Analysis Effect Size (LefSE) biomarker-based analysis comparing taxonomic abundances between healthy, seborrheic, and lesional sample types at visit 1 (**C**). Relative abundance (%) (**left**) and LDA scores (**right**) of significantly varying taxa (FDR *p*-value < 0.05).

Unique and shared ASVs between lesional, healthy, and seborrheic samples were identified and characterized at the phyla and genera taxonomic levels for both visit 1 and visit 2. Of note, in both visits, 39% of the total ASVs for the three sample types were shared ASVs. Of the unique ASVs, lesional samples consisted of 11% and 12% of the total ASVs (visits 1 and 2, respectively), as compared to 19% and 15% of the total ASVs for healthy samples (visits 1 and 2, respectively) and 15% and 19% of the total ASVs for seborrheic samples (visits 1 and 2, respectively). At the phyla taxonomic level, Firmicutes, Proteobacteria, and Actinobacteria were the predominant ASVs shared between the three sample types in both visit 1 and visit 2 (Figure 2A and 2B, respectively). At the genera taxonomic level, for the ASVs shared between the sample types, the predominant genera (all with more than 8% relative abundance) included *Staphylococcus*, *Acinetobacter, Corynebacterium*, *Streptococcus*, and *Enhydrobacter* in both visit 1 and visit 2 (Figure S4). Of the ASVs unique to lesions, *Staphylococcus*, *Acinetobacter*, and *Corynebacterium* were the more predominant (all more than 3% relative abundance), while ASVs unique to healthy or seborrheic samples were predominantly *Streptococcus*, *Acinetobacter*, *Corynebacterium,* and *Rothia* (all with more than 7% relative abundance).

### 3.6. LefSE Biomarker-Based Differential Abundance between Sample Types

LefSE biomarker-based analysis was conducted comparing sample types at visit 1 (Figure 2C) and visit 2 (Figure S5). A significantly lower relative abundance of the families *Neisseriaceae*, *Pasteurellaceae*, and *Leptotrichiaceae* was observed in lesional skin samples compared to healthy and seborrheic samples in visit 1. In comparison, a significantly higher relative abundance of *Staphylococcus* was observed in the lesional samples compared to the healthy and seborrheic samples in visit 2.

### 3.7. Taxonomic Abundance

**Figure 3:**
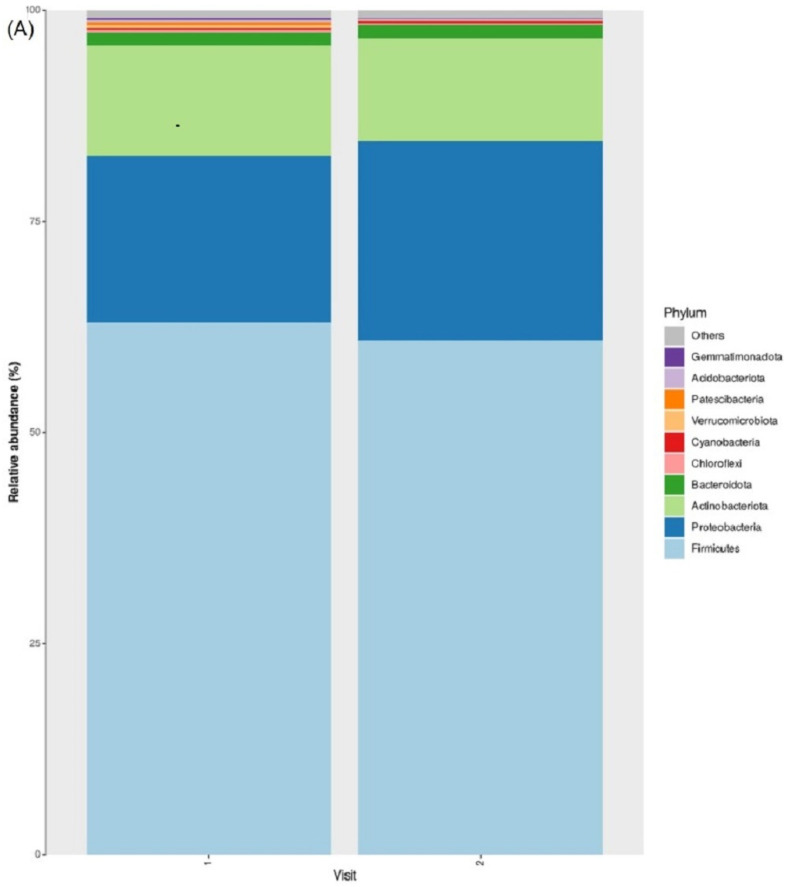

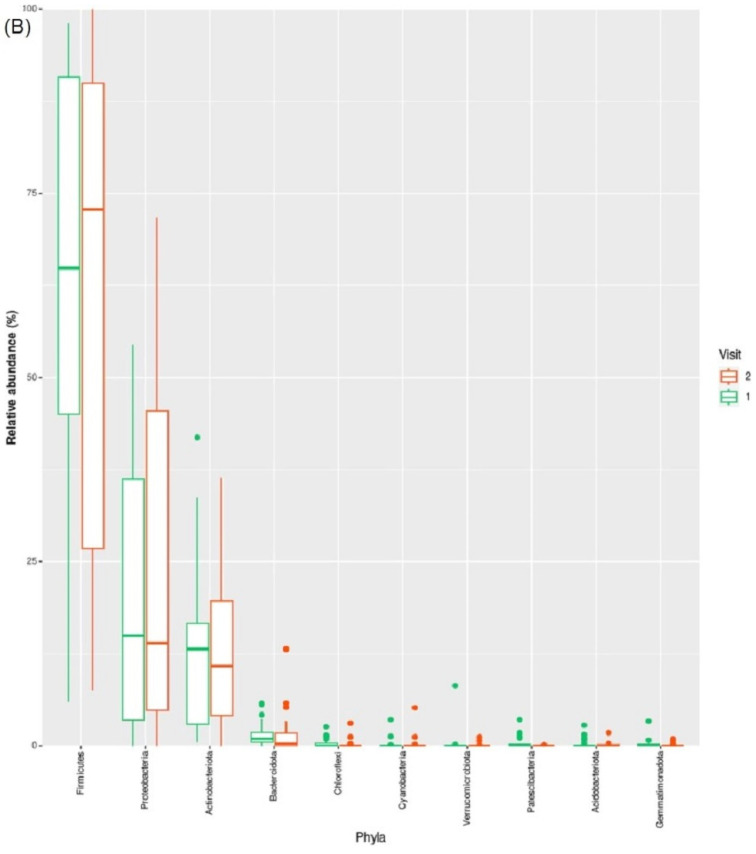

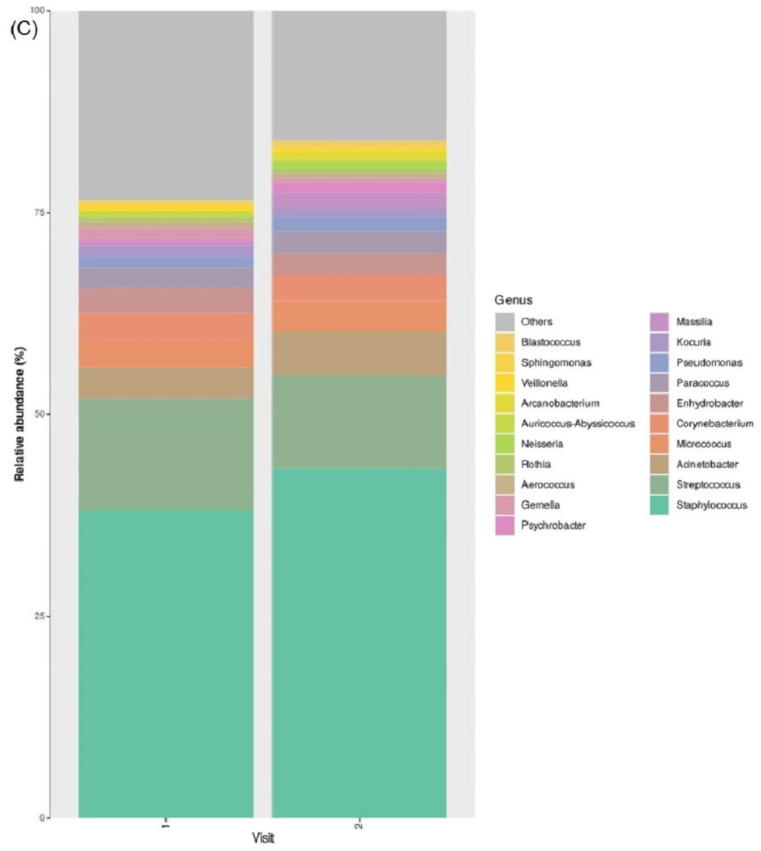

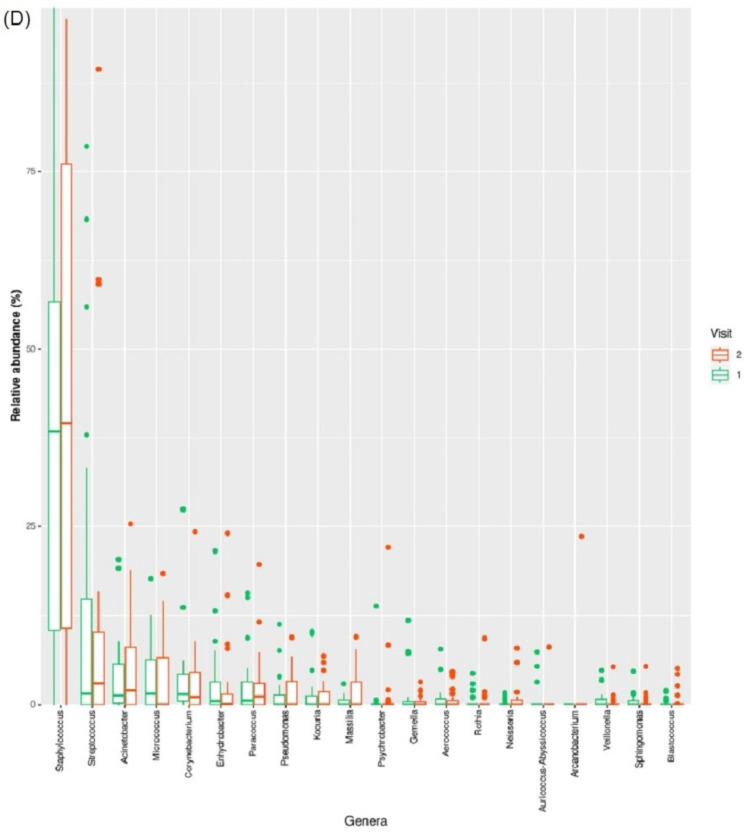
Abundant Bacteria in the Cutaneous Lesional EB Microbiome: Top 10 most abundant phyla in lesional samples at visit 1 (*n* = 25) and visit 2 (*n* = 25). (**A**,**B**) comparison of each phyla between visits. A statistically significant decrease in Patescibacteria abundance between visits was observed (Welch’s paired *t*-test, FDR *p*-value < 0.05). Top 10 most abundant genera in lesional samples at visit 1 (*n* = 25) and visit 2 (*n* = 25). (**C**,**D**) comparison of each genus between visits. A statistically significant (Welch’s paired *t*-test, FDR *p*-value < 0.05) decrease in *Dietzia* abundance between visits was observed, while a statistically significant increase in *Massilia* abundance between visits was observed.

Dominant phyla included Firmicutes, Proteobacteria, and Actinobacteriota (Figure 3A,B), with Firmicutes (means 63% and 61% for visit 1 and visit 2, respectively) and Actinobacteriota (means 13% and 12% for visit 1 and visit 2, respectively) decreasing in abundance over time, while Proteobacteria (means 20% and 24% for visit 1 and visit 2, respectively) increased in abundance over time. Notably, a statistically significant change was observed only for Patescibacteria (means 0.4% and 0.01% for visit 1 and visit 2, respectively, Welch’s paired *t*-test, FDR *p*-value < 0.05).

Dominant genera include *Staphylococcus*, *Streptococcus*, and *Acinetobacter* (Figure 3C,D), with *Staphylococcus* (means 38% and 43% for visit 1 and visit 2, respectively) and *Acinetobacter* (means 3.8% and 5.3% for visit 1 and visit 2, respectively) increasing in abundance over time, while *Streptococcus* (means 13.8% and 11.6% for visit 1 and visit 2, respectively) decreased in abundance over time. Notably, a statistically significant change was observed for *Dietzia* (decrease over time, means 0.3% and 0.01% for visit 1 and visit 2, respectively, Welch’s paired *t*-test, FDR *p*-value < 0.05) and *Massilia* (increase over time, means 0.4% and 1.8% for visit 1 and visit 2, respectively, Welch’s paired *t*-test, FDR *p*-value < 0.05).

### 3.8. Alpha Diversity

**Figure 4:**
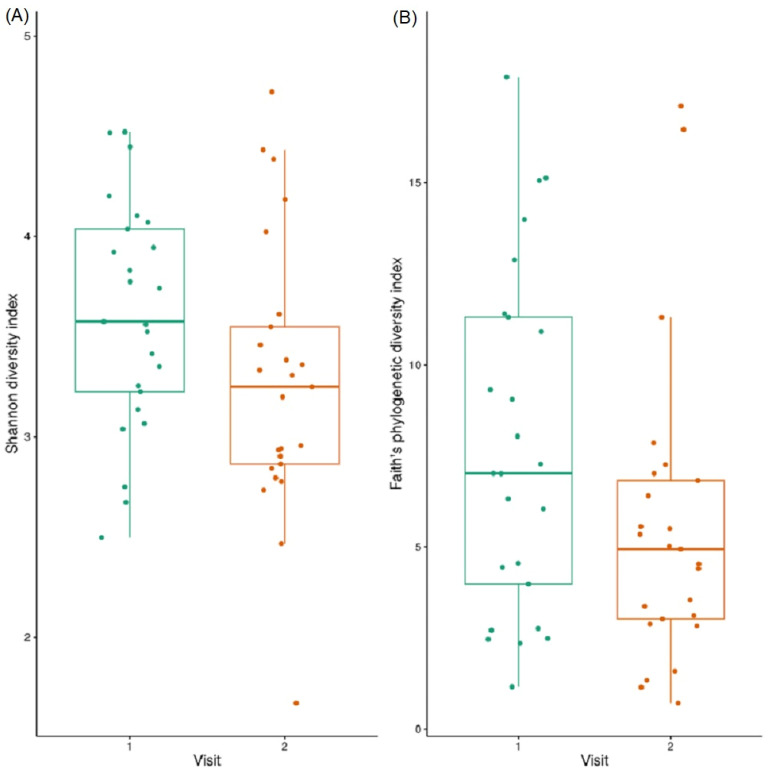

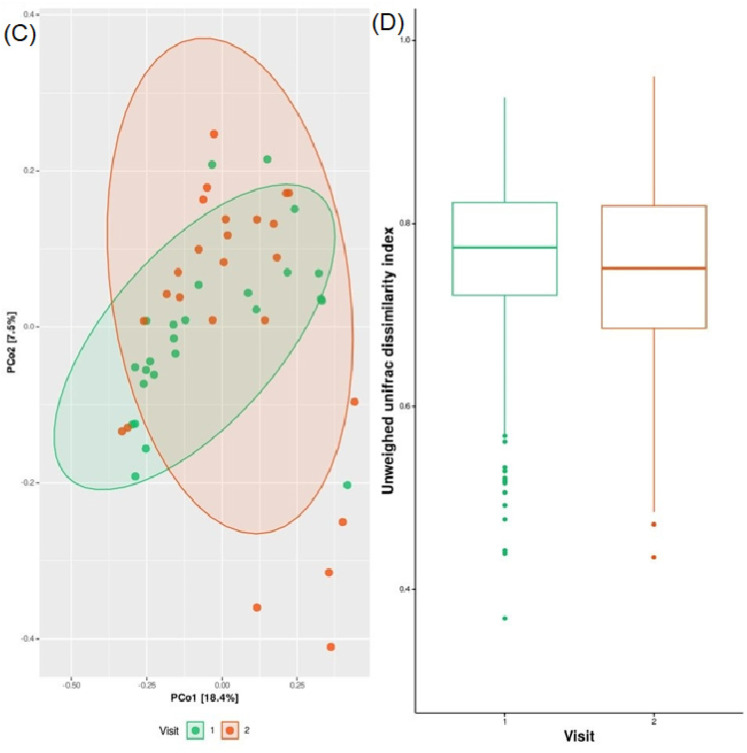
Temporal Dynamics of the Cutaneous Lesional EB Microbiome. Alpha diversity analysis of lesional samples at visit 1 and visit 2. No significant differences (paired Welch’s *t*-test with FDR) were observed using both (**A**) Shannon diversity index, and (**B**) Faith’s phylogenetic diversity indices, though a trend of decrease in diversity measures between visits was noted. Principal coordinates analysis (PCoA) plot of lesional samples at visit 1 and visit 2, with confidence ellipses of beta diversity analysis (Unweighted UniFrac dissimilarity index) of lesional samples at visit 1 and visit 2. (**C**) PERMANOVA (999 permutations) between lesional samples at visit 1 and visit 2 did not show a statistically significant (FDR *p*-value < 0.05) difference between the visits (**D**).

No significant differences (Welch’s paired *t*-test) were observed between the lesional skin samples over time (using both Shannon diversity and Faith’s phylogenetic diversity indices). However, both measures of alpha diversity showed a trend of decrease in diversity over time (Figure 4A,B).

### 3.9. Beta Diversity

No significant differences (PERMANOVA [999 permutations]) were observed between the lesional samples over time (using both Bray-Curtis and Unweighted Unifrac dissimilarity indices, Figure 4C,D), though both measures of beta diversity showed a trend of increase in similarity between the samples over time.

The effect of sampling locations (i.e., dry, moist, and sebaceous) on variations in taxonomic composition between lesional and non-lesional samples was observed both at the phyla and genera taxonomic levels. The Firmicutes phylum was significantly more dominant in the dry lesional samples as compared to the dry non-lesional samples (means 62% and 37%, respectively; Wilcoxon, FDR *p*-value < 0.05), while the phyla Proteobacteria and Actinobacteria were more dominant in the dry non-lesional samples as compared to the dry lesional samples, with a significant difference observed for Proteobacteria (means 38% and 21%, respectively; Wilcoxon, FDR *p*-value < 0.05) (Figure S6A). At the genus level, *Staphylococcus* and *Streptococcus* were more dominant in the dry lesional samples as compared to the dry non-lesional samples, with significant differences observed for *Staphylococcus* (means 42% and 17%, respectively; Wilcoxon, FDR *p*-value < 0.05), while *Acinetobacter* was significantly more dominant in the dry non-lesional samples as compared to the dry lesional samples (means 13% and 5%, respectively; Wilcoxon, FDR *p*-value < 0.05) (Figure S6B).

## 4. Discussion

This study characterized the skin microbiome of pediatric patients with EB by comparing skin lesions to healthy skin and observing microbiome population dynamics over time following treatment courses. We reveal several novel insights into lesion-driven dysbiosis with potential therapeutic implications. Lesional skin samples harbored significantly less *Bacteroidota* and *Fusobacteriota* prior to the initiation of treatment, whereas following topical dressing, we observed more *Firmicutes* and less *Proteobacteria* in lesion samples when compared to both healthy and seborrheic samples. The beta diversity differences in bacterial composition between sites are evident when observing Bray-Curtis dissimilarity both prior to and following treatment, whereas alpha diversity differences, as measured by the Shannon index, are only evident following treatment. LefSe analysis indicated that *Staphylococcus* was significantly more abundant in lesional samples than in healthy and seborrheic samples following treatment. Whereas no significant differences were noted between sampling sessions for both alpha and beta diversity metrics, we did observe an increase in *Staphylococcus* and *Acinetobacter* spp. between sampling sessions at the expense of *Streptococcus*.

Previous culture-independent studies on the EB microbiome have shown reduced bacterial diversity and increased staphylococcal carriage when compared to healthy controls, both of which are more pronounced in lesional skin than healthy skin [18] and on the skin of patients with severe disease as opposed to intermediate disease [19]. Our study is the first to document the temporal dynamics of the skin microbiome in patients with EB, and interestingly, our results are in agreement with the previously published studies at the second sampling session but not the first. We surmise that the diseased state drives a consistent dysbiosis of the skin microbiome, as documented in the two previously published studies [18,19], whereas topical dressings drive local temporal changes. To buttress our hypothesis, we note that *S. aureus* tends to colonize moist areas of the skin in a healthy state [4], and the moisture-rich environment provided by the topical dressings may support *S. aureus* outgrowth.

Previous culture-based studies have documented the colonization of EB chronic wounds by *Staphylococcus* spp., consistent with our findings [20,21,22]. Interestingly, we detected *Staphylococcus* spp. at all sampled sites, including on the samples taken from sites where EB was not clinically active. However, *Staphylococcus* spp. abundance on wounds was most striking on sebaceous sites (Supplementary Materials Figure S6A,B), potentially owing to the altered physiochemical characteristics of the site due to EB activity.

Van der Kooi-Pol et al. [21] described multiple *Staphylococcus* spp. colonization of the skin of patients with EB, as opposed to a singular strain colonizing the skin, which is often the case in healthy and persistent *S. aureus* carriers. Elsewhere [23], they documented a high degree of genetic diversity of *S. aureus* strains colonizing EB patients. A limitation of our study is the 16S methodology, which does not provide the taxonomic resolution necessary to revisit Van der Kooi-Pol et al.’s observation; therefore, future studies using whole genome metagenomics sequencing are needed. Similarly, longitudinal stability or instability of the *S. aureus* strains colonizing the chronic wounds may provide insight as to the clinical course of patients with EB.

Similar to Fuentes et al. [24], we observed reduced microbial diversity on chronic wound sites when compared to non-wounded sites and increased *Staphylococcus* spp. colonization in the skin of EB patients. Whereas Fuentes et al. demonstrated this on two separate small cohorts with recessive dystrophic EB, we did so on a larger cohort of 32 patients and a broader swath of EB types. These two observations were reported on a separate cohort of eight patients with dystrophic EB using 16S sequencing methodology. Another potential limitation of our study was the relatively small number of dystrophic EB patients; therefore, the strength of our conclusions may not be equally identical for both simplex and dystrophic subtypes. Nevertheless, our study, coupled with the previous reports of *S. aureus* carriage in EB skin, adds to the growing body of literature which associates *S. aureus* colonization of the skin with cutaneous diseases [25]. The inclusion of a larger group of rare EB variants is a challenge that should be addressed in future studies.

## 5. Conclusions

Our study recaptured the reduced bacterial diversity and increased staphylococcal carriage noted by others in EB patients. This was observed only following topical treatment and not preceding topical treatment, indicating a potential effect of topical dressing either directly on the wound microbiome or indirectly through the contribution towards skin healing. The detection of *Firmicutes* in general, and *S. aureus* specifically, commensurate with the application of a wound dressing may warrant the use of additional treatment methods to facilitate wound healing. Additional important findings of our study include the increased relative abundance of *Proteobacteria* in lesional sites, consistent beta diversity dissimilarity between healthy and lesional skin at both visits, and alpha diversity differences between sites following the application of topical dressings. Further studies using whole genome metagenomic sequencing have the potential to uncover the strain-level differences both before and after treatment in this population. Additionaly, further studies in these patients should prospectively correlate the temporal changes in the microbiome associated with various treatment modalities in order to optimize the care of EB patients.

## Figures and Tables

**Table 1:** Demographics, clinical and genetic features of the study cohort (*n* = 32).

Patient No.	Age (Years)	Sex	Ethnicity	EB Type	Diagnosis	Genetic Mutation
1	16	Female	Jewish	Simplex localized	Clinical	N/A
2	5	Female	Bedouin	Simplex localized	Clinical	N/A
3	7	Male	Bedouin	Simplex localized	Clinical	N/A
4	11	Female	Bedouin	Simplex localized	Clinical	N/A
5	9	Male	Bedouin	Simplex localized	Clinical	N/A
6	7	Male	Bedouin	Simplex localized	Clinical	N/A
7	9	Female	Bedouin	Simplex localized	Clinical	N/A
8	6	Male	Bedouin	Simplex localized	Clinical	N/A
9	3	Male	Bedouin	Simplex localized	Clinical	N/A
10	0.1	Male	Bedouin	Simplex localized	Clinical	N/A
11	2	Male	Bedouin	Simplex localized	Clinical	N/A
12	7	Male	Bedouin	Simplex localized	Clinical	N/A
13	14	Male	Bedouin	Simplex localized	Clinical	N/A
14	6	Male	Bedouin	Simplex localized	Clinical	N/A
15	5	Male	Bedouin	Simplex localized	Clinical	N/A
16	2	Female	Bedouin	Simplex localized	Clinical	N/A
17	2	Female	Bedouin	Simplex localized	Clinical	N/A
18	3	Male	Bedouin	Simplex localized	Clinical	N/A
19	5	Female	Bedouin	Simplex localized	Clinical	N/A
20	4	Male	Bedouin	Simplex localized	Genetic	KRT14
21	1	Female	Bedouin	Simplex generalized	Genetic	KRT14
22	13	Male	Bedouin	Simplex generalized	Genetic	KRT14
23	0.9	Male	Jewish	Simplex generalized	Genetic	KRT14
24	1	Female	Bedouin	Simplex generalized	Genetic	KRT14
25	0.1	Male	Bedouin	Simplex generalized	Genetic	KRT14
26	1	Male	Bedouin	Simplex generalized	Genetic	KRT14
27	4	Male	Bedouin	Simplex generalized	Genetic	PLEC
28	1	Female	Jewish	Simplex generalized	Genetic	PLEC
29	0.5	Male	Bedouin	Dystrophic recessive	Genetic	COL7A
30	2	Male	Bedouin	Dystrophic recessive	Genetic	COL7A
31	2	Male	Bedouin	Dystrophic recessive	Genetic	COL7A
32	0.6	Male	Bedouin	Dystrophic recessive	Genetic	COL7A

## Data Availability

The clinical data are not publicly available due to ethical restrictions. We have full control of all primary data and agree to allow the journal to review the data if requested. Further enquiries can be directed to the corresponding author. 16S *rRNA* amplicon sequencing data are available under BioProject PRJEB66184.

## Supplementary Materials

The following supporting information can be downloaded at: https://www.mdpi.com/article/10.3390/jcm12206435/s1, Figure S1. Relative abundance of phyla that showed significant differences (KW, FDR *p*-value < 0.05) between healthy, seborrheic, and lesional sample types at (A) visit 1 and (B) visit 2. Figure S2. Relative abundance of families that showed significant differences (KW, FDR *p*-value < 0.05) between healthy, seborrheic, and lesional sample types at (A) visit 1 and (B) visit 2. Figure S3. Relative abundance of genera between healthy, seborrheic, and lesional sample types at (A) visit 1 and (B) visit 2. Note that no significant differences were observed at (A) visit 1, while (B) visit 2 showed significant differences (KW, FDR *p*-value < 0.05). Figure S4. Mean relative abundances of top 16 genera from the unique and shared ASVs between healthy, seborrheic, and lesional sample types (from the Venn diagram in Figure 4A,B), that were observed in both visit 1 and visit 2. Figure S5. Linear Discriminant Analysis Effect Size (LefSE) biomarker-based analysis comparing taxonomic abundances between healthy, seborrheic, and lesional sample types at visit 2. Relative abundance (%) (left) and LDA scores (right) of significantly varying taxa (FDR *p*-value < 0.05). Figure S6. Comparison of Bacteria on Lesional and non-Lesional Sites: (A) Mean abundances at phylum and genus taxonomic levels of non-lesional samples at both visits in different sampling locations, namely, dry (*n* = 53), moist (*n* = 1), and sebaceous (*n* = 54). (B) Mean abundances at phylum and genus taxonomic levels of lesional samples at both visits in different sampling locations, namely, dry (*n* = 54), moist (*n* = 1), and sebaceous (*n* = 1).
