# Supplementary figures and images for: Temporal Changes in the Skin Microbiome of Epidermolysis Bullosa Patients following the Application of Wound Dressings
Source: J Clin Med. 2023 Oct 10;12(20):6435. doi: 10.3390/jcm12206435 (PMC10607196; doi:10.3390/jcm12206435)

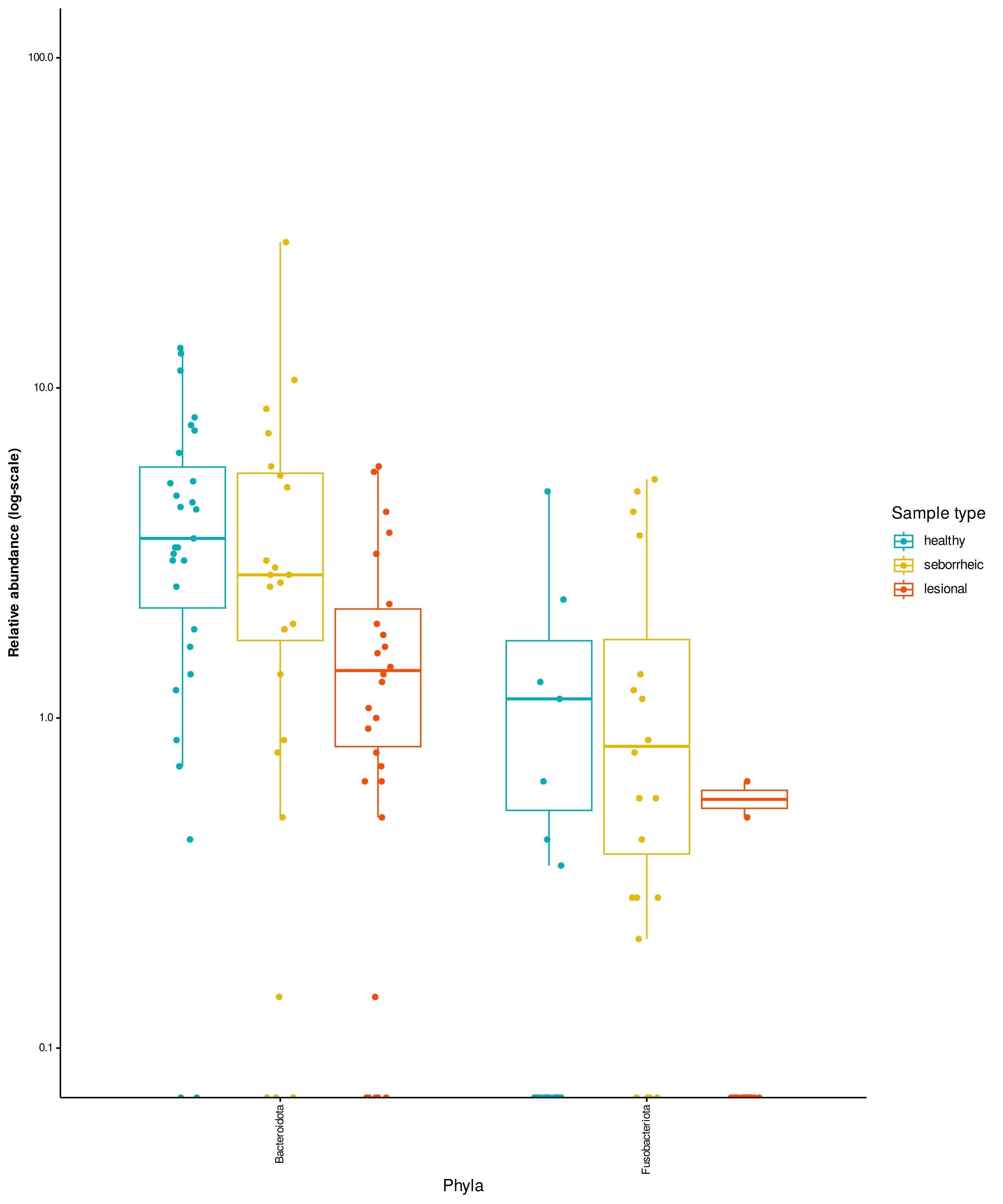

Supplement: Supplementary file 1 [file jcm-12-06435-s001.zip › figureS1A.jpg]

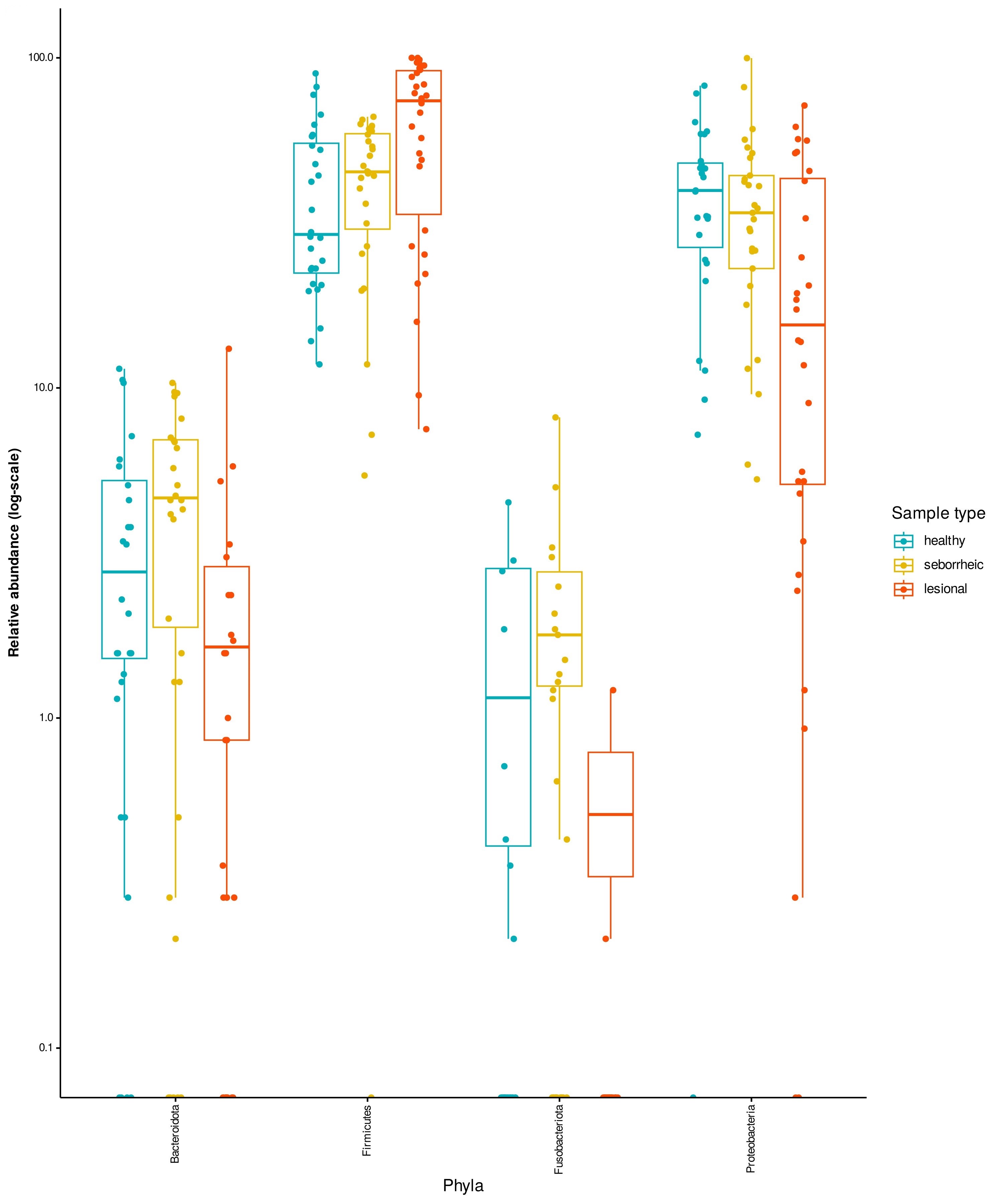

Supplement: Supplementary file 1 [file jcm-12-06435-s001.zip › figureS1B.jpg]

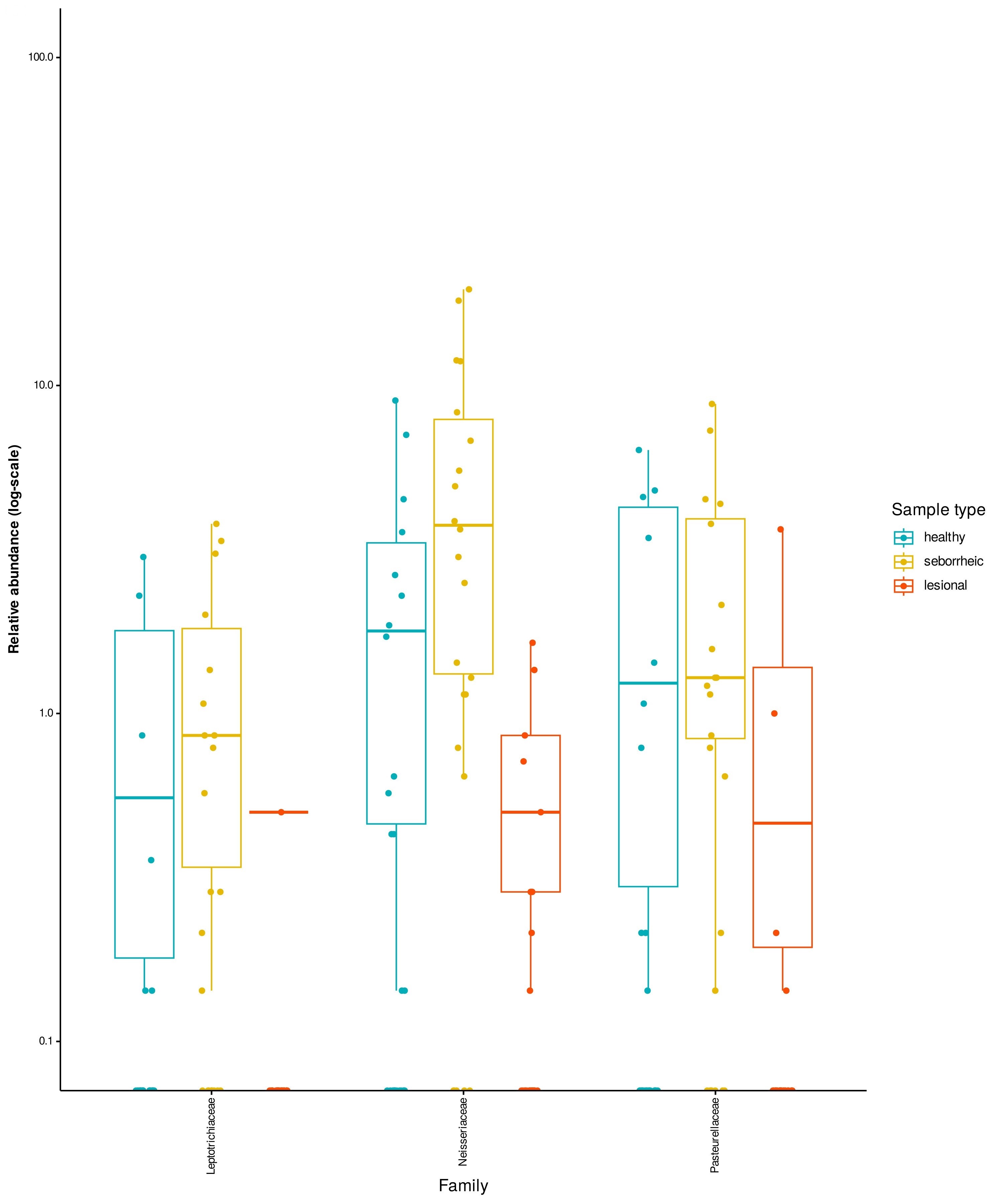

Supplement: Supplementary file 1 [file jcm-12-06435-s001.zip › figureS2A.jpg]

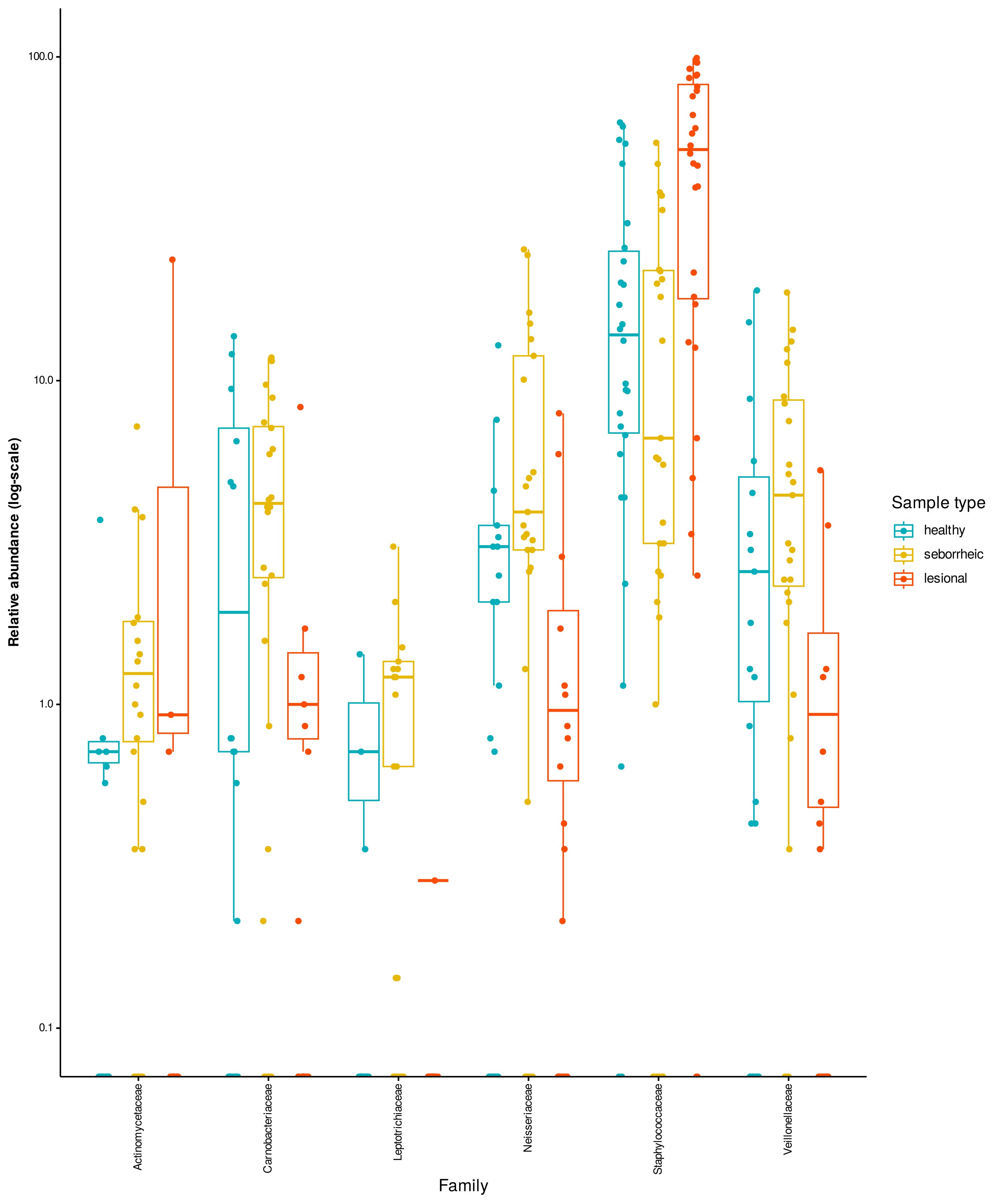

Supplement: Supplementary file 1 [file jcm-12-06435-s001.zip › figureS2B.jpg]

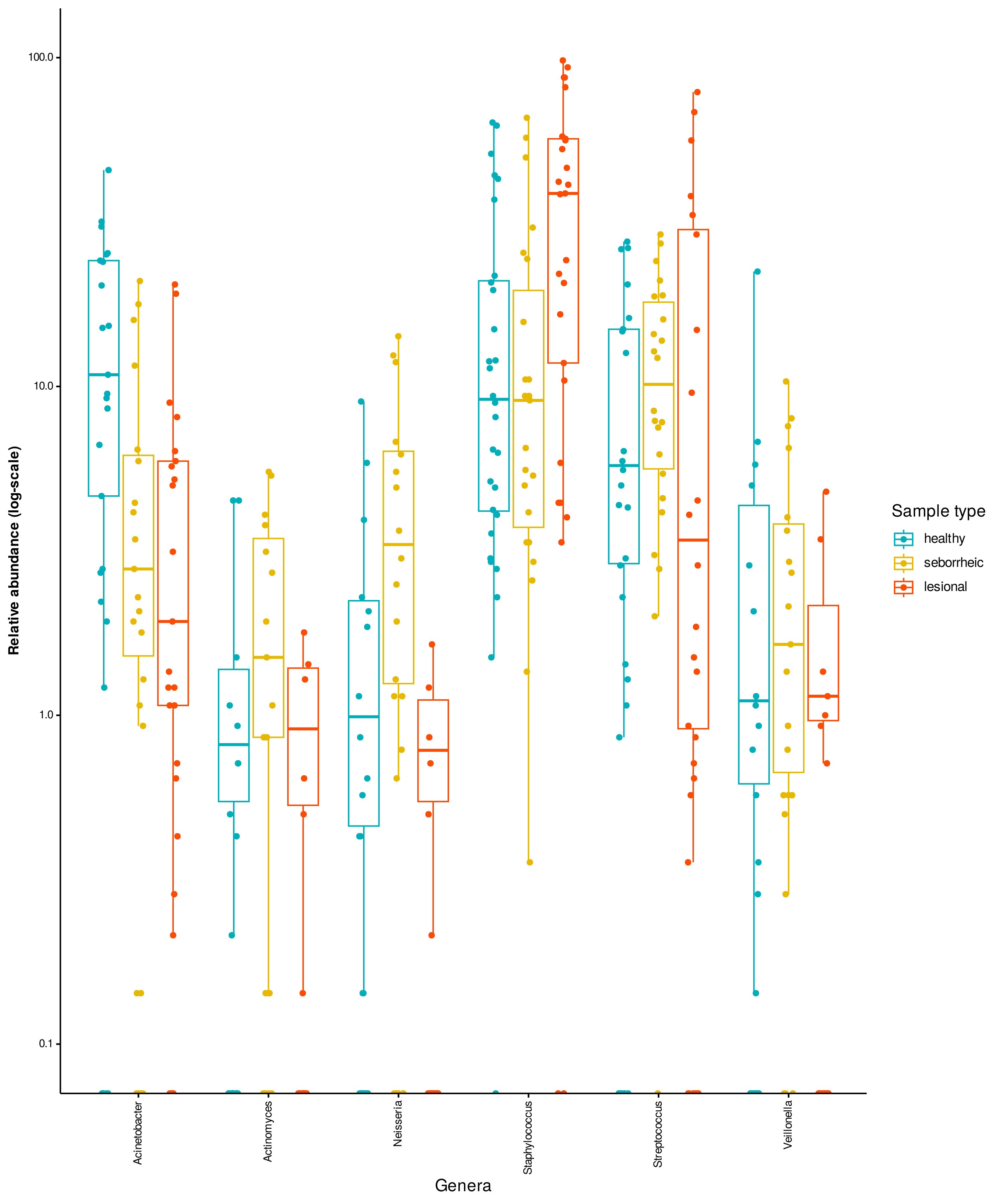

Supplement: Supplementary file 1 [file jcm-12-06435-s001.zip › figureS3A.jpg]

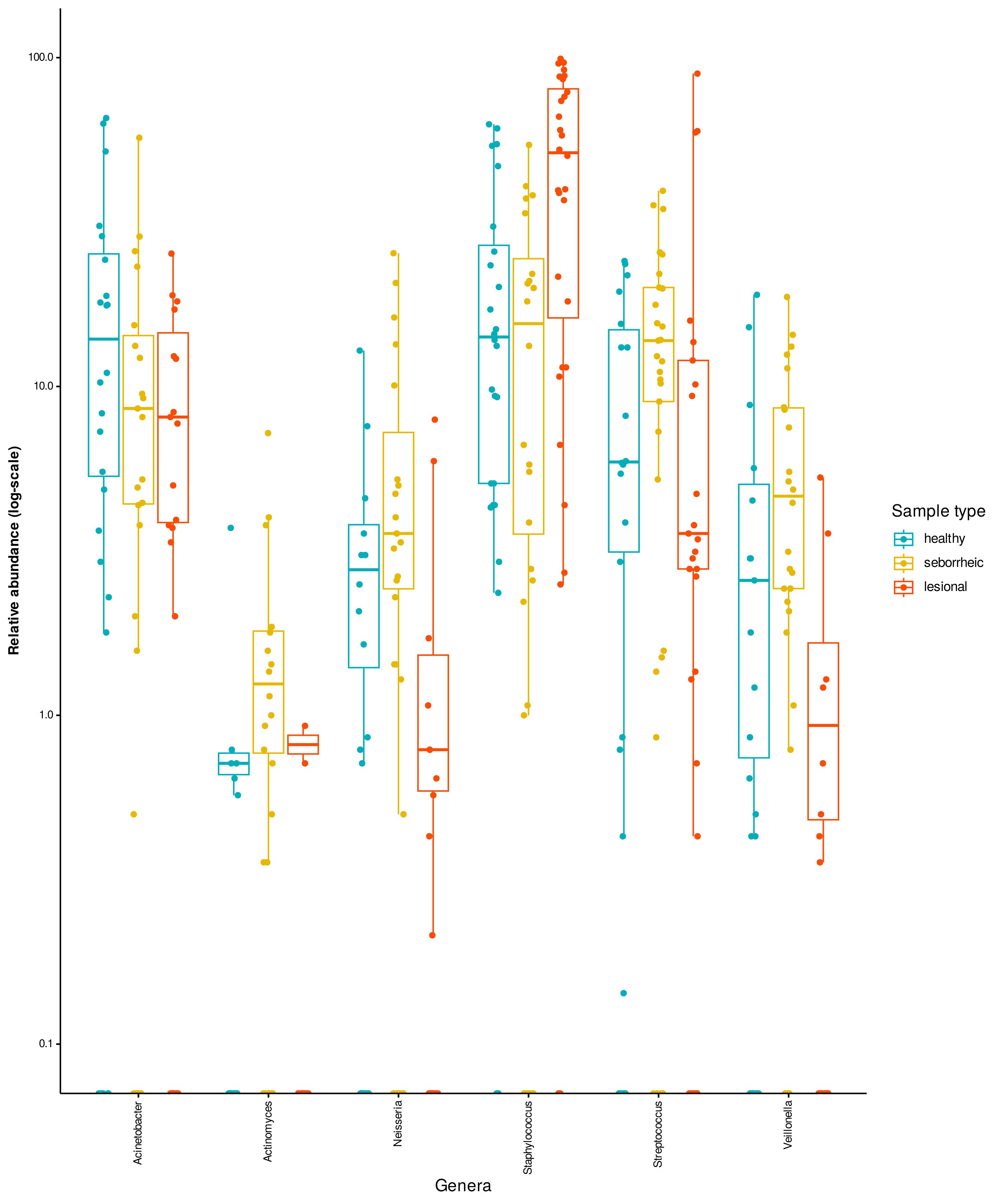

Supplement: Supplementary file 1 [file jcm-12-06435-s001.zip › figureS3B.jpg]

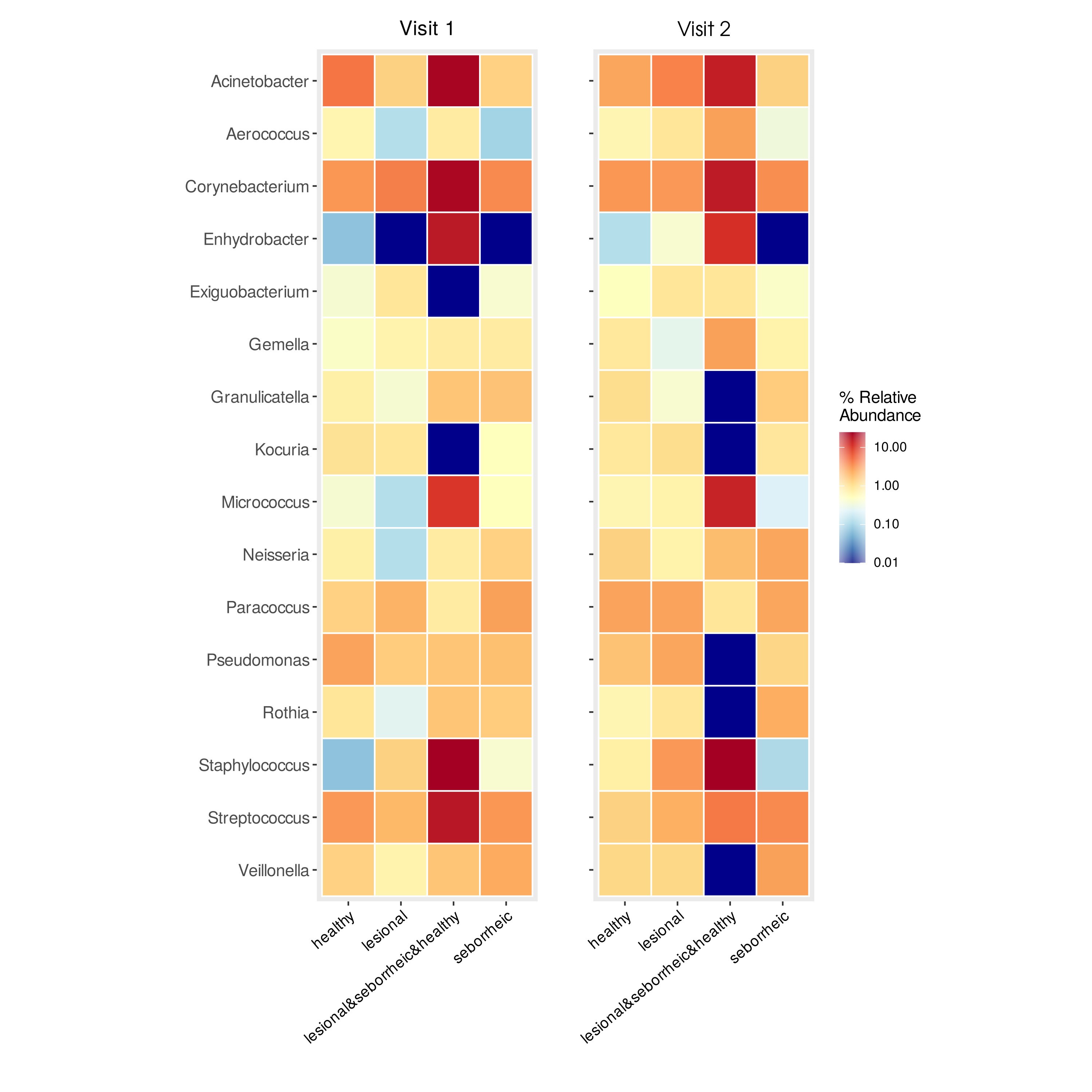

Supplement: Supplementary file 1 [file jcm-12-06435-s001.zip › figureS4.jpg]

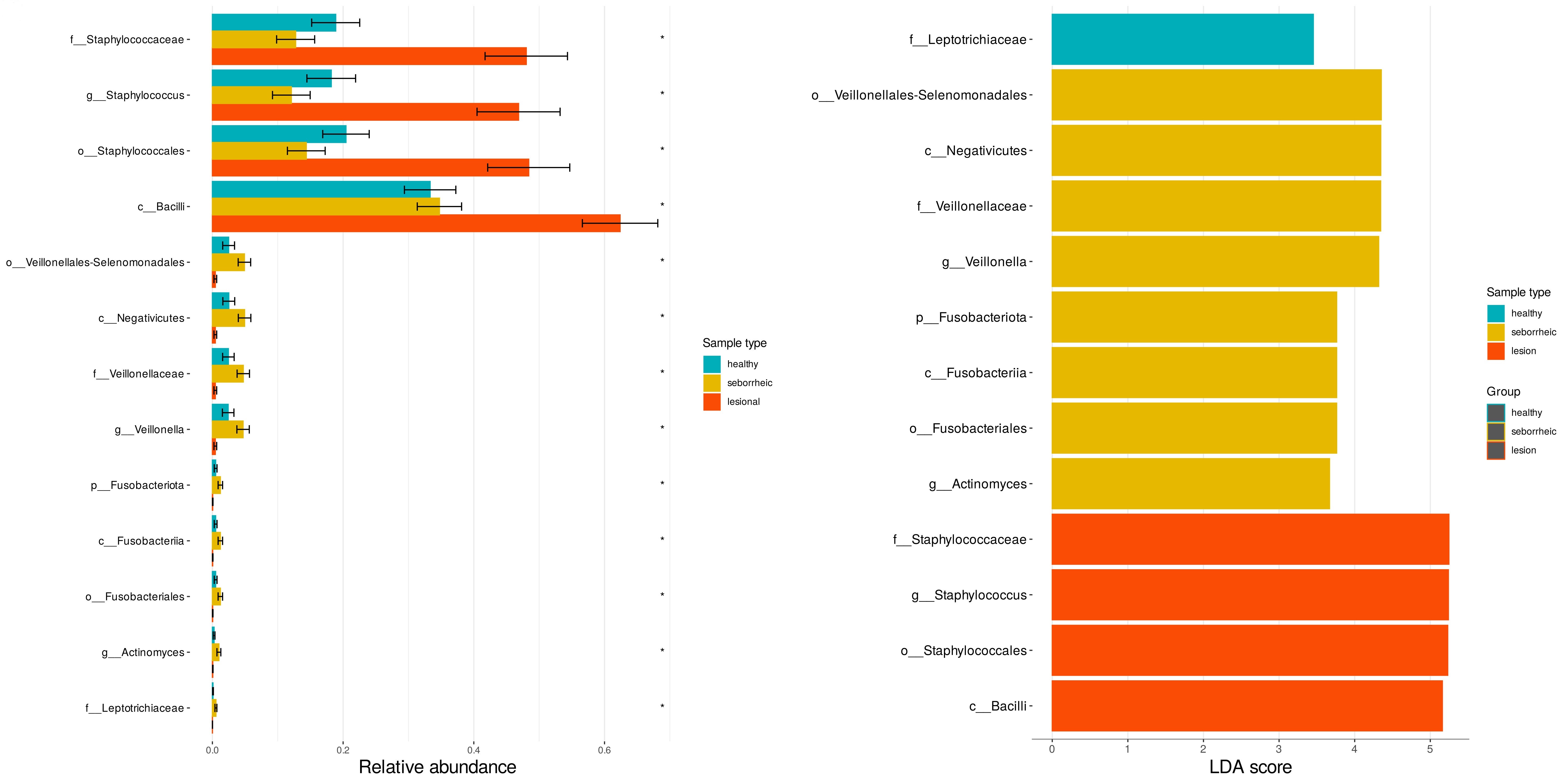

Supplement: Supplementary file 1 [file jcm-12-06435-s001.zip › figureS5.jpg]

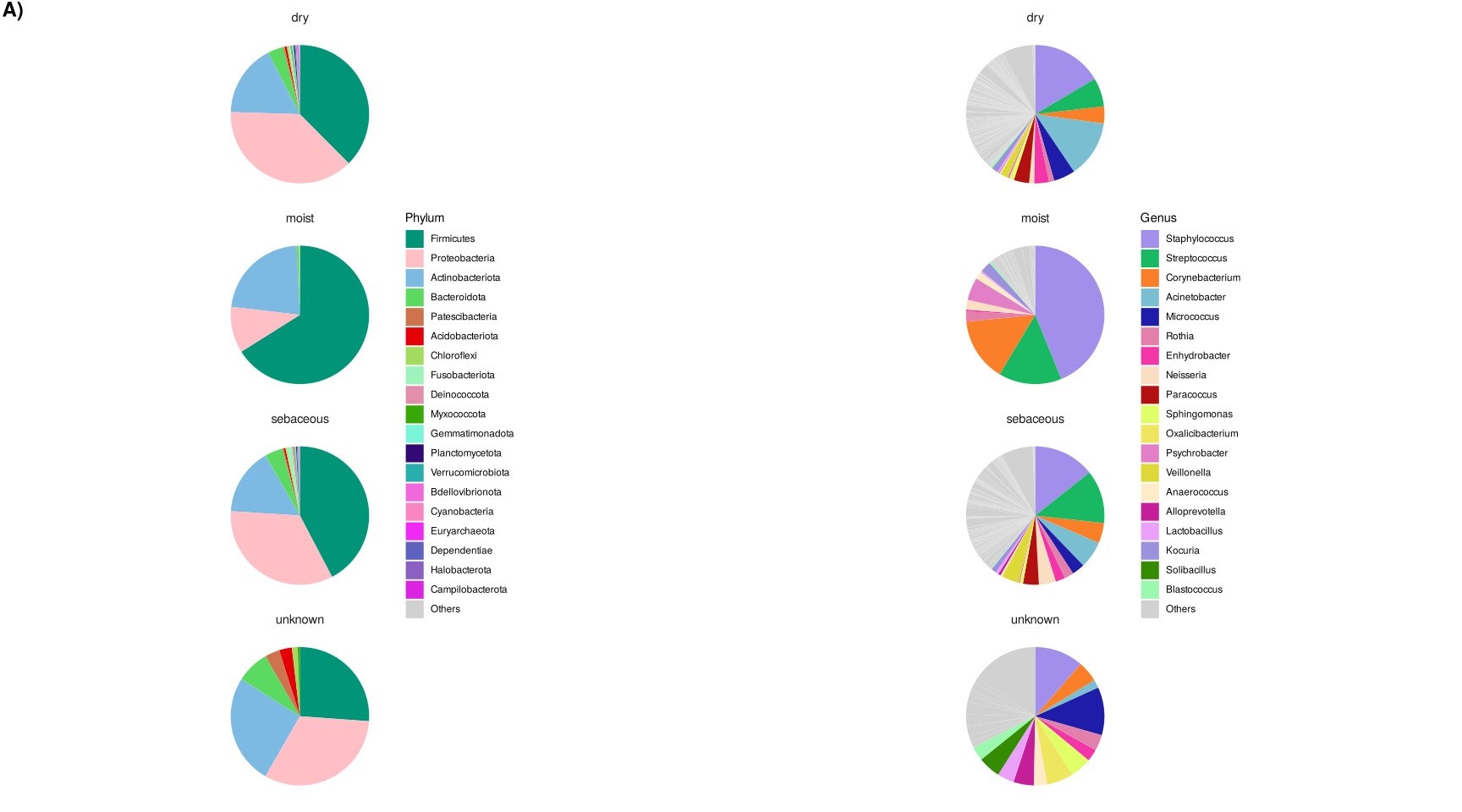

Supplement: Supplementary file 1 [file jcm-12-06435-s001.zip › figureS6A.jpg]

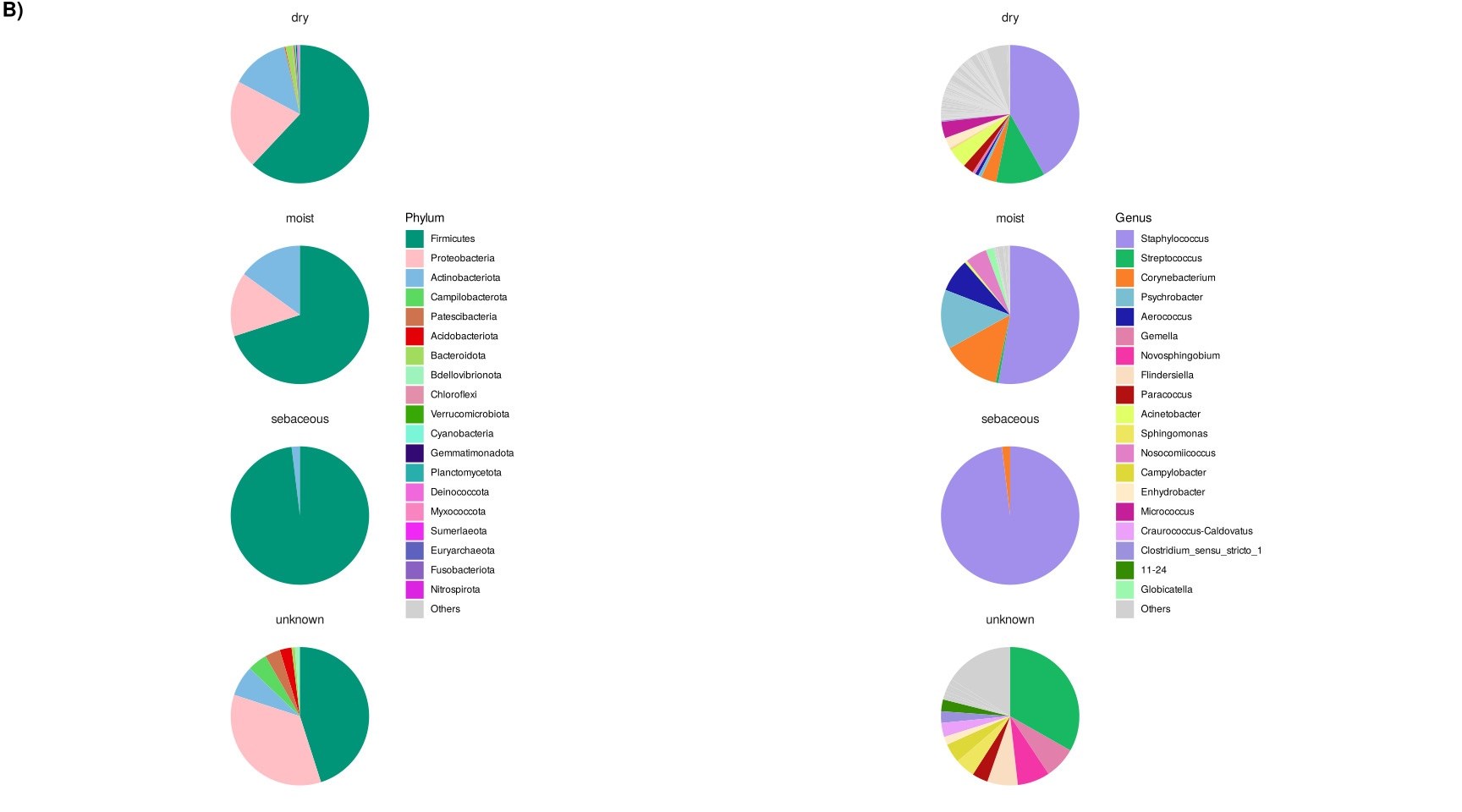

Supplement: Supplementary file 1 [file jcm-12-06435-s001.zip › figureS6B.jpg]
